# The impact of social determinants of health on obesity and diabetes disparities among Latino communities in Southern California

**DOI:** 10.1186/s12889-022-14868-1

**Published:** 2023-01-06

**Authors:** Joseph C. Cleveland, Juan Espinoza, Elizabeth A. Holzhausen, Michael I. Goran, Tanya L. Alderete

**Affiliations:** 1 Department of Integrative Physiology, University of Colorado Boulder, Boulder, CO USA; 2 Department of Pediatrics, The Saban Research Institute, Children’s Hospital Los Angeles, University of Southern California, Los Angeles, CA USA

**Keywords:** Social Determinants of Health, Latino, Healthy Places Index, Social Vulnerability Index, CalEnviroScreen, Childhood Obesity, Obesity, Type 2 Diabetes

## Abstract

**Background:**

Social determinants of health (SDoH) describe the complex network of circumstances that impact an individual before birth and across the lifespan. SDoH contextualize factors in a community that are associated with chronic disease risk and certain health disparities. The main objective of this study was to explore the impact of SDoH on the prevalence of obesity and diabetes, and whether these factors explain disparities in these health outcomes among Latinos in Southern California.

**Methods:**

We utilized three composite indices that encompass different SDoH: the Healthy Places Index (HPI), Social Vulnerability Index (SVI), and CalEnviroScreen (CES). Univariate linear regression models explored the associations between index scores with adult obesity, adult diabetes, and childhood obesity.

**Results:**

Communities with lower HPI scores were associated with higher prevalence of metabolic disease and a greater proportion of Latino residents. Cities in the lowest decile of HPI scores had 71% of the population identifying as Latino compared to 12% in the highest decile. HPI scores explained 61% of the variability in adult obesity (*p* < 0.001), 41% of the variability in childhood obesity (*p* < 0.001), and 47% of the variability in adult diabetes (*p* < 0.001). Similar results were observed when examining SVI and CES with these health outcomes.

**Conclusions:**

These results suggest that Latinos in Southern California live in communities with adverse SDoH and face a greater burden of adult obesity, diabetes, and childhood obesity.

**Supplementary Information:**

The online version contains supplementary material available at 10.1186/s12889-022-14868-1.

## Background

In the United States, communities of color, under-resourced, and marginalized communities experience significant disparities in metabolic health, including obesity, type 2 diabetes, and cardiovascular disease [1, 2]. For example, approximately 84% of Latino adults have overweight or obesity compared to 74% non-Hispanic white adults [3]. Disparities in obesity begin in early childhood; the prevalence of obesity in Latino pre-school children is nearly 5 times higher than in non-Hispanic whites [4]. Additionally, certain characteristics of the built environment result in disadvantaged communities living in more obesogenic environments [5]. Acknowledging disparities in childhood obesity is critical because this time period is important for obesity prevention [6] and obesity is a leading risk factor for other chronic diseases such as asthma [7, 8], cardiovascular disease [9], and type 2 diabetes [10]. In addition to the physical complications associated with excess adiposity, individuals with obesity are 1.55 times more likely to report clinically significant depressive symptoms [11]. There is also growing evidence of a bidirectional association between obesity and type 2 diabetes with depression, suggesting that depressive symptomology may compound the future burden of disease [12, 13]. Collectively, these multiple adverse health outcomes contribute to a shorter life expectancy, increased healthcare costs, and decreased lifetime earning potential, all of which further contribute to increased vulnerability [14–16].

The socioecological model of obesity provides a conceptual framework to understand the complex interplay between person-level factors, such as behavior and physiology, with larger social, cultural, economic, and environmental factors [17, 18]. Collectively, these contextual factors are known as social determinants of health (SDoH), defined as the “conditions in which people are born, grow, live, work, and age” [19]. Studies have shown that individuals with greater access to greenspace have lower rates of diabetes [20]. Additionally, communities with greater access to supermarkets and limited access to convenience stores have lower levels of obesity [21]. Furthermore, adults with diabetes and food insecurity are 40% more likely to have poor glycemic control [22]. Therefore, there is an urgent need to understand the collective impact of SDoH on chronic disease risk and associated health disparities. For this reason, several composite indices have been created, including the California Healthy Places Index 2.0 (HPI) [23], the Social Vulnerability Index 2018 (SVI) [24], and the California EnviroScreen 4.0 (CES) [25]. Of these, the HPI is the most recent tool, originally published in 2018 by the Public Health Alliance of Southern California, and was designed to help prioritize disadvantaged communities in public policy and investments [26].

These composite indices are made up of various SDoH indicators but differ in the way they weight community-level attributes. For example, in the HPI, economic factors are given the most weight while housing, healthcare access, and environmental exposures are given less weight. This is compared to the CES, which equally weighs environmental exposures and population characteristics. Lastly, the SVI equally weighs socioeconomic, household composition, minority status, and housing factors. While other studies have found these individual indices are associated with life expectancy at birth [26], COVID infection rates [27], or heat-related injury [28], no studies have examined the associations between these indices and the burden of metabolic disease in Latino communities in California. This is noteworthy since California has the largest population of Latinos and the third largest percent Latino population nationwide [29]. Compared to the state average of 39%, Southern California has a higher percent Latino population with approximately 10.6 million Latino residents, comprising a total of 45% of the population [29].

The overall aim of this study was to determine the relationships between SDoH (as represented by the HPI) with certain metabolic diseases (e.g., obesity, diabetes) in Latino communities in Southern California. We hypothesized that adverse SDoH would be associated with a greater burden of metabolic disease, especially among Latino communities. Given that the HPI is a relatively new indicator that more heavily weighs economic factors, we sought to compare the associations between HPI and health outcomes with the SVI and CES. Finally, we sought to examine the associations between SDoH (as represented by the HPI) and health outcomes with known disparities linked to poor metabolic health, such as poor mental health and prevalence of adults with asthma.

## Methods

This is an ecological study that examined census-tract level scores for ten contiguous counties in Southern California. This analysis relied on publicly available and de-identified data. The Institutional Review Board provided an Official Determination that this analysis does not meet the definition of human subject research. Thus, institutional review and approval was not required.

### Data sources

#### Composite indices

At the time of analysis, we utilized the most recent version of each composite index, including the HPI 2.0, SVI 2018, and CES 4.0. The Public Health Alliance of Southern California created the HPI, which is composed of 25 indicators organized into eight policy domains (Supplemental Table 1) [23]. Each of the 25 indicators are standardized using a z-score and then combined, based on its component weight, into the index score. Each indicator is adjusted to scale in the same direction, meaning lower values correspond to more adverse SDoH present in a community. The HPI 2.0 uses data from 2010 to 2015 and we examined HPI as a percentile ranging from 0 to 100, with 100 indicating the healthiest community conditions. The Centers for Disease Control and Prevention (CDC) created the SVI 2018 to describe the relative vulnerability of census tracts and to identify communities in need of support during emergencies. The SVI includes 15 indicators grouped into four equally weighted domains that aggregate to the overall vulnerability score (Supplemental Table 2). The directionality of the SVI score is opposite to the HPI score. A higher SVI score indicates greater vulnerability, and a lower score indicates less vulnerability. CalEnviroScreen 4.0 was developed by the Office of Environmental Health Hazard Assessment to identify a community’s vulnerability to environmental pollutants. This index uses 21 indicators grouped into two equally weighted categories (Supplemental Table 3). These two categories are comprised of four subdomains: exposures, environmental effects, sensitive populations, and socioeconomic factors. The environmental effect subdomain is given half the weight of the exposure subdomain in the pollution burden score and the sensitive population subdomain and socioeconomic factor subdomain are equally weighted in the population characteristics score. The pollution burden score and population characteristics scores are equally weighted in the aggregate CES score. Like the SVI, the CES score has the opposite directionality to the HPI score. A higher CES score indicates higher vulnerability to environmental pollutants and a lower score indicates lower vulnerability.

#### Adult health outcomes

In addition to HPI scores, the Public Health Alliance of Southern California collects decision support indicators that are made available to be used in conjunction with the HPI [23]. These decision support indicators are grouped into various domains, and we examined nine indicators in the health outcomes domain. These nine decision support indicators are sourced from the CDC 500 Cities Project, the California EnviroScreen 3.0, and the Virginia Commonwealth University (Supplemental Table 4). The primary analysis focused on two adult health outcomes, diabetes prevalence and obesity prevalence. Briefly, diabetes prevalence was estimated from the percentage of respondents 18 or older who self-reported being told by a health professional they had diabetes. Diabetes included both type 1 and type 2 diabetes but did not include gestational diabetes. Obesity prevalence was based on BMI values derived from self-reported height and weight values from respondents 18 or older [30]. The secondary analysis examined health outcomes with known associations with poor metabolic health, including prevalence of poor mental health, poor physical health, current asthma, current smoking [30], life expectancy at birth [23], asthma ER admissions, and heart attack ER admissions [31]. The HPI 2.0 was published in 2018, and it contains indicators and health outcome data from 2010 to 2015.

#### Childhood obesity

Available school-level body composition data was sourced from the California Department of Education 2019 Physical Fitness Test and is presented in Supplemental Table 4. The Physical Fitness Test includes a series of tests for 5th, 7th, and 9th grade students administered annually in California schools. This testing series has three options to measure body composition, body mass index, skinfold measurements, and bioelectric impedance analyzer. Based on these measures, body composition results were grouped into four zones: very lean, healthy fitness zone, needs improvement, and needs improvement-health risk. The number of students in each zone was reported at the school level. For the purposes of this analysis, students in the needs improvement-health risk zone were characterized as having obesity. The needs improvement health-risk zone is an adequate proxy for children with obesity because this bottom threshold for this zone aligned with a BMI at approximately the 95th percentile for age and sex specific childhood obesity measures published by the CDC [32].

### Data transformation

Index scores, including HPI, SVI, and CES, were reported as percentiles at the census tract level. The HPI assigns each census tract to a city. To perform our analysis at the city level, we took the simple average of percentiles for all census tracts within the same city. We chose to examine the simple average of percentiles because a population-weighted methodology would heavily favor the densest counties and would result in lack of resolution to examine the variability in HPI scores. However, we also compared a population-weighted and simple average calculation for HPI and found that results were consistent (Supplemental Table 5). For uniformity, we mapped the census tract level data from the SVI and CES to the city level assignments in the HPI. All health outcomes, except for childhood obesity, were reported as percentiles at the census tract level based on relative prevalence. We applied the same methodology as the index scores and took the simple average of percentiles for all census tracts within the same city. Finally, percent Latino was reported at the census tract level. Like the indices and health outcomes, we took the simple average of percent Latino for all census tracts within the same city, according to the HPI assignments. There are several terms used in the literature that describe the community of focus, such as, Latino, Hispanic, and Latinx. In the HPI, the Public Health Alliance of Southern California describes the indicator variable as “percent Latino”. Thus, to be consistent with our data source, we chose to exclusively use the term Latino to describe the community of focus [23]. For the purposes of this analysis, higher average percentiles for each health outcome indicates relatively poorer health (e.g., higher prevalence of obesity relative to other cities examined). Four cities were excluded from this analysis given a lack of health data: Bradbury, Irwindale, and Vernon (all in Los Angeles County); and Sunset Beach in Orange County.

Childhood obesity was examined as an average percentile at the city level. The California Department of Education 2019 Physical Fitness Test provides latitudes and longitudes for each school, which we mapped to census tracts using Texas A&M University GeoServices. To be consistent with the other health outcomes examined, we calculated percentiles based on relative obesity prevalence at the census tract level. From the census tract level, we used the city assignments provided by the HPI to calculate a simple average of childhood obesity percentiles for all census tracts in the same city.

### Statistical analysis

Descriptive statistics were performed to examine the mean and standard deviations for the raw scores of each index. We utilized univariate linear regression models to investigate the associations between the percentile of each index with the percentile of adult obesity, childhood obesity, adult diabetes, and each additional health outcome. We also examined how these associations covary with percent Latino. Our regression analyses examined variables at the city level because we wanted to examine associations of city level SDoH with the health outcomes of interest. However, we also performed our analysis at the census tract and county levels and found similar results (Supplemental Fig. 1). For all analyses, we report the R^2^ values and corresponding *p*-values. While the percentiles for each index and each health outcome were examined, the raw scores for each index are presented in Table 1. Statistical significance was based on a two-side p-value of 0.05. All statistical analyses were performed in R (Version 1.4.1717).

**Table 1:** Descriptive Statistics

***Composite Indices***	Number of Cities (***n***)	Value (Mean ± SD)	Percent Latino (Mean ± SD)
**Healthy Places Index 2.0 (HPI)**			
All Cities with HPI Scores	367	−0.04 ± 0.46	39.0% ± 25.1%
HPI Score + Childhood Obesity Prevalence Data	341	− 0.03 ± 0.45	40.1% ± 25.3%
HPI Score + Adult Diabetes and Obesity Prevalence Data	162	−0.06 ± 0.40	42.4% ± 22.8%
**Social Vulnerability Index (SVI)**			
All Cities with SVI Scores	371	0.49 ± 0.25	39.1% ± 25.4%
SVI Score + Childhood Obesity Prevalence Data	344	0.49 ± 0.24	40.1% ± 25.4%
SVI Score + Adult Diabetes and Obesity Prevalence Data	162	0.51 ± 0.23	42.4% ± 22.8%
**CalEnviroScreen 4.0 (CES)**			
All Cities with CES Scores	370	26.8 ± 14.6	39.0% ± 25.3%
CES Score + Childhood Obesity Prevalence Data	343	27.3 ± 14.7	40.1% ± 25.4%
CES Score + Adult Diabetes and Obesity Prevalence Data	162	28.9 ± 13.6	42.4% ± 22.8%

This table displays the raw, unadjusted scores for the HPI, SVI, and CES. HPI scores range from − 1.15 to 1.09 and a higher score corresponds to healthier community conditions. The HPI scores for the subset of cities that had data available for adult diabetes, adult obesity, and childhood obesity are included as well as the Percent Latino for the 367 cities with HPI data and the subset of cities with data available on the relevant health outcomes. SVI scores range from 0.017 to 0.99 and a higher SVI score corresponds to a less healthy city. Like the SVI, CES scores range from 3.93 to 68.52 and higher scores correspond to less healthy cities

## Results

### HPI, SVI, and CES city level scores

**Fig. 1:**
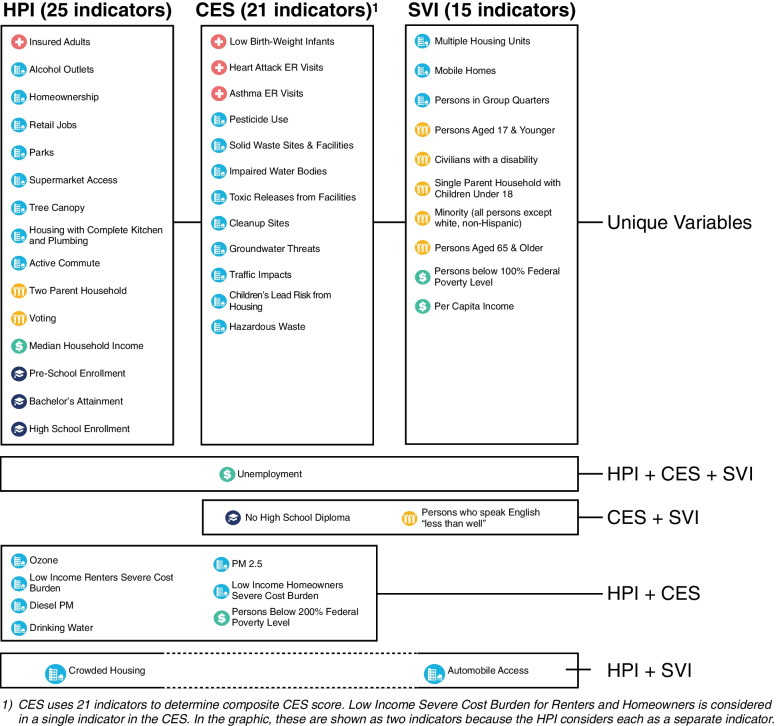
Comparison of SDoH Represented in HPI, CES, and SVI. This figure highlights each of the SDoH indicators captured by the HPI, CES, and SVI. From top to bottom, the figure highlights the indicators that are specific to each index, the indicators shared by two indices, and the indicators shared by all three indices

**Fig. 2:**
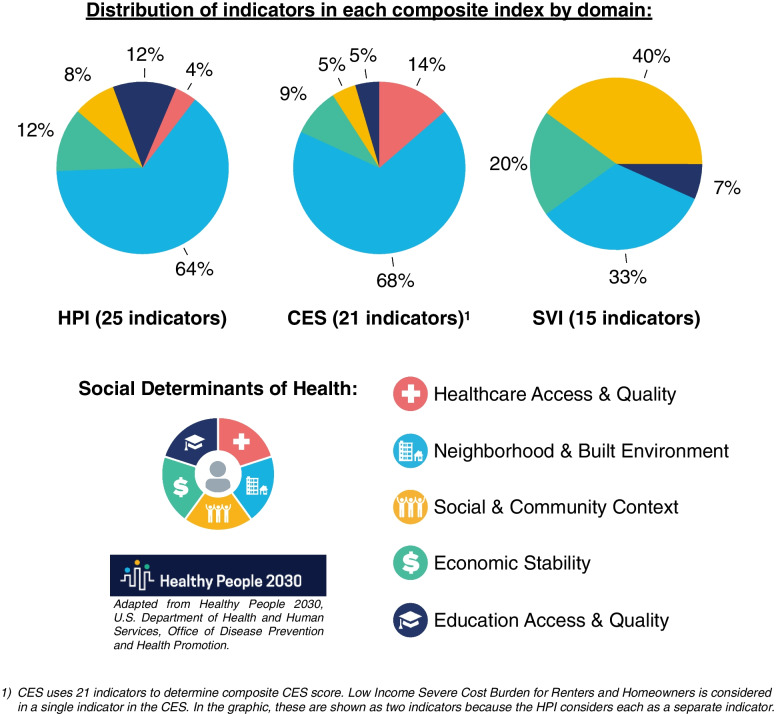
Distribution of SDoH Represented in HPI, CES, and SVI. At the top of the figure, the distribution of the SDoH domains in each index is presented. At the bottom of the figure, the domains in which SDoH are categorized are indicated

As shown in Figs. 1 and 2, HPI, SVI, and CES rely on several shared indicators. However, the number of unique indicators for each index outnumber the shared indicators, which highlights that each index uses different methodology to weigh various SDoH. Despite this, the CES and HPI both have a similar distribution of *Neighborhood and Built Environment* indicators relative to other SDoH domains. Lastly, SVI has the greatest number of *Social and Community Context* indicators, which is a domain that is not as well represented in the distribution of HPI and CES indicators.

The raw scores for each index are presented in Table 1. Briefly, HPI scores range from − 1.15 to 1.09 with higher scores corresponding to healthier city conditions (mean HPI: − 0.04). For the SVI and the CES, a higher score indicates less healthy community conditions and SVI scores ranged from 0.017 to 0.99 (mean SVI: 0.49) and CES scores ranged from 3.93 to 68.52 (mean CES: 26.8). Not all cities had available data for adult obesity and diabetes; however, the mean HPI, SVI, and CES scores among these cities was comparable to the mean of all Southern California cities (Table 1). The average prevalence of adult obesity in the cities included in this analysis was 25.1%, which was slightly higher than the 2014 state-wide obesity prevalence of 24.7% [33]. The average diabetes prevalence was 10.2%. This mean prevalence was slightly higher than the 2014 California diabetes prevalence of 9.9% [34].

### Latinos are exposed to less healthy community conditions

**Fig. 3:**
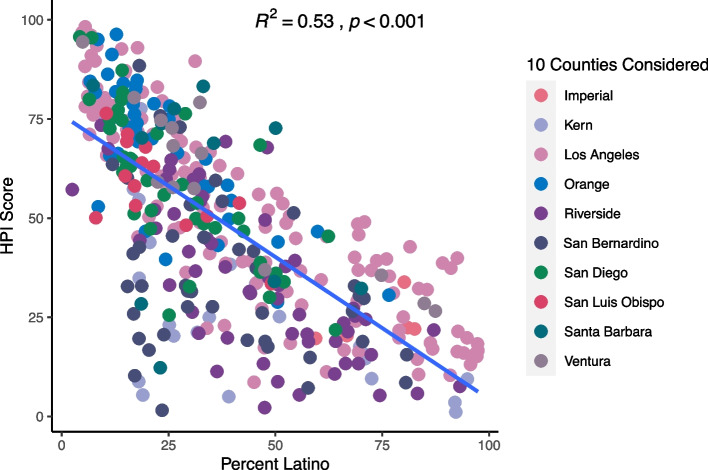
Southern California Communities with a Higher Proportion of Latino Residents had a Lower HPI Score. Each point on the scatterplot represents one of the 367 Southern California cities included in the analysis. Cities were grouped into counties and are displayed using distinct colors. The x-axis represents the percent Latino within each of the Southern California cities. The y-axis represents the HPI Score Percentile, with 0 representing the community with the least healthy community conditions and 100 representing the community with the healthiest community conditions

**Fig. 4:**
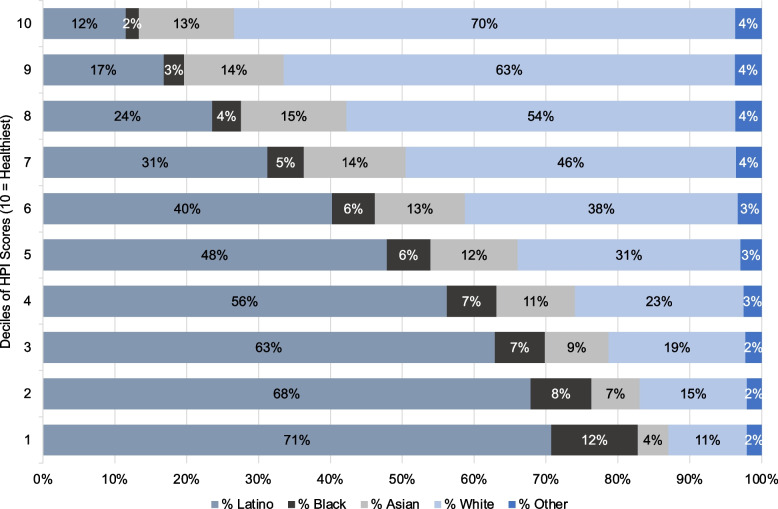
Cities with Lower HPI Scores Have Higher Percent Latino Populations than Cities with Higher Scores. Each bar on this graph represents a decile of city-level HPI scores. Each number label corresponds to the percent race/ethnicity in each of the deciles. The top decile (10) indicates the decile with the highest HPI scores and the decile with the bottom decile (1) indicates the decile with the lowest HPI scores. As demonstrated in the figure above, increasing deciles of disadvantage (i.e., going down the graph) have a notably higher percent Latino population

As shown in Fig. 3, we observed a strong inverse association between the percentile of the HPI score (higher score = healthier community conditions) and percent Latino in the Southern California region (R^2^ = 0.53; *p* < 0.001). Similar patterns of association were observed when we examined the SVI and CES scores (Supplemental Figs. 2 & 3), which indicate that multiple adverse SDoH, including environmental exposures, aggregate in Latino communities in Southern California. As shown in Table 2, counties with less healthy HPI scores had a higher prevalence of each adverse outcome investigated, which included poor physical health, poor mental health, and chronic asthma. We also found that increasing deciles of disadvantage had corresponding increases in the percent of the population identifying as Latino (Fig. 4).

**Table 2:** Less Healthy Communities Face Greater Burden of Adverse Health Outcomes, Adverse Health Behaviors, and Shorter Life Expectancy

				% Prevalence in county						Rate of ER Visits per 10,000		
County	HPI Score	HPI Score Percentile	Latino (%)	Diabetes	Obesity	Mental Health Not Good	Physical Health Not Good	Current Asthma	Current Smoker	Asthma ER Admissions	Heart Attack ER Admissions	Life Expectancy (years)
Imperial	− 0.50	24.4%	75.4%	–	–	–	–	–	–	88.9	11.9	81.8
Kern	−0.42	29.3%	47.0%	10.4%	30.5%	14.2%	14.4%	9.3%	18.7%	64.2	11	77.4
San Bernardino	−0.32	33.1%	48.3%	10.7%	27.6%	13.9%	14.6%	9.3%	17.1%	67.0	11.6	78.7
Riverside	−0.26	35.9%	44.2%	10.2%	28.8%	13.4%	13.9%	9.1%	17.0%	47.1	10.3	79.5
Los Angeles	−0.14	42.6%	46.9%	11.0%	26.3%	13.1%	14.1%	8.5%	15.8%	51.6	8.3	81.5
San Diego	0.09	54.7%	31.7%	9.4%	23.0%	11.9%	11.6%	8.2%	14.3%	40.7	6.2	81.2
Santa Barbara	0.19	60.9%	38.2%	9.5%	24.4%	12.5%	13.1%	8.8%	14.8%	38.4	5.6	81.5
San Luis Obispo	0.20	61.7%	19.5%	–	–	–	–	–	–	32.5	5.2	81.0
Orange	0.20	61.0%	31.3%	9.0%	21.0%	11.5%	11.6%	8.1%	14.2%	32.0	6.8	82.3
Ventura	0.21	61.6%	37.8%	9.2%	24.0%	11.7%	12.1%	8.5%	13.9%	37.6	8.0	81.8

This table shows the metabolic health indicators that were strongly associated with the HPI score based on R^2^ values. The unadjusted values (i.e., not percentiles) are shown for each health indicator in each of the ten counties examined. Except for life expectancy, an increase in prevalence corresponds to a greater disease burden. Asthma ER and Heart Attack ER Admissions are presented as a rate per 10,000 in population

### Less healthy community conditions were associated with a higher percentile of obesity and diabetes

**Fig. 5:**
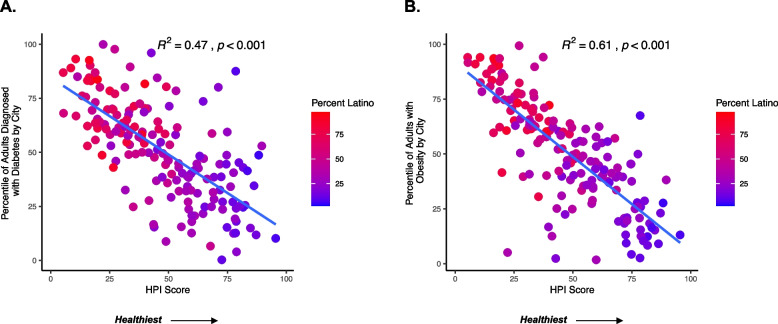
Less Healthy Environments are Associated with a Greater Prevalence of Obesity and Diabetes. Each dot on the scatterplot represents a city within the ten counties investigated in this analysis. The x-axis shows the percentile HPI score, and a higher percentile score indicates a healthier community. The y-axis shows the percentile score of the prevalence of each health outcome relative to each other (i.e., a percentile score of 100 translates to the city with the highest prevalence of adult diabetes or obesity). Each city on the scatterplot shows a gradation of the percent Latino in each city. A red shading indicates a higher percent Latino compared to a purple shading. Panel A shows the relationship between adults with diabetes (excluding gestational diabetes) and HPI score. Panel B shows the relationship between adults with obesity and HPI score

**Fig. 6:**
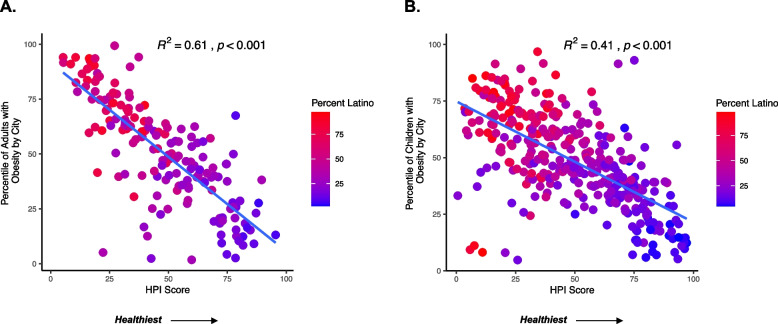
Less Healthy Environments are Associated with a Greater Prevalence of Childhood Obesity. Each point on the scatterplot represents a city within the ten counties investigated in this analysis. The x-axis shows the percentile HPI score, and a higher percentile score indicates a healthier community. The y-axis shows the percentile score of the prevalence of each health outcome relative to each other (i.e., a percentile score of 100 translates to the city with the highest prevalence of adult or childhood obesity). Each point (i.e., city) on the scatterplot is colored based on the percent Latino in each city. A red shading indicates a higher percent Latino compared to a purple shading. Panel A shows the relationship between adults with obesity and HPI score. Panel B shows the relationship between children with obesity and HPI score. 162 cities had available adult obesity data compared to 341 cities with available childhood obesity data

The HPI percentile score explained 61% of the variability in adult obesity (*p* < 0.001) and 47% of the variability in adult diabetes (*p* < 0.001), indicating that less healthy community conditions were strongly associated with the prevalence of certain metabolic diseases (Fig. 5). We also found that cities with a higher percentile of each metabolic disease had a higher proportion of Latino residents and some of the lowest HPI scores. Given that obesity originates early in life, [35] we next examined the relationship between HPI and childhood obesity and found that a lower HPI percentile score (less healthy community conditions) explained 41% of the variability in childhood obesity (*p* < 0.001) (Fig. 6). Overall, cities with a higher percentile of childhood obesity had lower HPI percentile scores and a higher proportion of Latino residents. Similar results were observed when examining the SVI and CES scores (Supplemental Figs. 4, 5, and 6). For example, we found that more vulnerable SVI percentile scores were associated with a higher percentile of adult obesity (R^2^ = 0.50, *p* < 0.001) and adult diabetes (R^2^ = 0.49, *p* < 0.001). We also found that communities with adverse environmental CES percentile scores had a higher percentile of adult obesity (R^2^ = 0.43, *p* < 0.001) and adult diabetes (R^2^ = 0.46, *p* < 0.001). Lastly, a less healthy SVI and CES percentile score explained 48% (*p* < 0.001) and 44% (*p* < 0.001) of the variability in childhood obesity, respectively.

### Less healthy community conditions were associated with greater disease burden and shorter life expectancy

Among the additional outcomes summarized in Table 3, a strong inverse association was observed in which a lower HPI percentile score (more community-level adverse SDoH) was associated with adverse health outcomes, especially in communities with a higher percentage of Latinos. For example, the HPI percentile score explained 72% of the variability in self-reported poor physical (*p* < 0.001) and 67% of the variability in poor mental health (*p* < 0.001). These findings indicate that Latinos live in environments with greater adverse SDoH and have a higher prevalence of poor physical and mental health, which are known to impact overall life expectancy [16, 36]. Indeed, the HPI score explained 42% of the variability in life expectancy (*p* < 0.001). As an example, Kern County, which has the second lowest average HPI score and third highest percent Latino population, had nearly a five-year lower life expectancy at birth compared to Orange County, which has the second highest average HPI score and the second lowest Latino population.

**Table 3:** Associations between HPI, CES, and SVI with Health Outcomes and Behaviors

Health Outcome	﻿R^**2**^		
	HPI*	CES*	SVI*
Adults 18+ Who Report 14+ Days During Past Month Which Physical Health was Not Good	0.72	0.52	0.64
Adults 18+ Who Report 14+ Days During Past Month Which Mental Health was Not Good	0.67	0.44	0.62
Adults 18+ Who Report Having Smoked 100+ Cigarettes in Lifetime and Currently Smoke Every Day or Some Days	0.61	0.34	0.52
Adults 18+ With BMI ≥ 30.0	0.61	0.43	0.50
Age Adjusted Rate of Emergency Dept Visits for Asthma Per 10,000	0.49	0.49	0.50
Adults 18+ Diagnosed with Diabetes (Excluding Gestational Diabetes)	0.47	0.46	0.49
Percent of Population Currently with Asthma	0.46	0.22	0.37
Life Expectancy at Birth	0.42	0.16	0.32
Children with Obesity	0.41	0.44	0.48
Rate of ER Visits for Heart Attacks (Per 10,000 ER Visits)	0.40	0.33	0.31

R^2^ values are from univariate linear regression models that examined the associations between each publicly available composite indices (HPI, CES, and SVI) with each health outcome or health behavior that has been linked with metabolic health. An asterisk (*) denotes that all univariate associations between each index and health outcome or behavior were statistically significant at a *p*-value < 0.001. Lastly, except for life expectancy, an increase in prevalence corresponds to a greater disease burden

## Discussion

In this study, we observed that adverse SDoH (as represented by HPI) were associated with a greater prevalence of adult obesity, adult diabetes, and childhood obesity among Latino communities. We found similar associations with the SVI and CES, two older and more established composite indices. Additionally, we found similar associations when examining the HPI using a simple average compared to a population weighted calculation. These findings suggest that the HPI methodology captures the burden of SDoH that contribute to less healthy conditions and a greater burden of metabolic disease in marginalized communities. Our observation that Latinos in Southern California live in communities with lower HPI scores also highlights the disproportionate impact of adverse SDoH on Latino communities in Southern California [37–39].

In our analysis, the HPI score explained a large percent of the variability in the percentile of adult obesity, adult diabetes, and childhood obesity, indicating that adverse SDoH are strongly associated with poor metabolic health. This is consistent with previous studies that have found that vulnerable communities (as represented by SVI) have a higher prevalence of overweight or obesity [40]. Similarly, other studies have found that food insecurity [41, 42], limited access to greenspace [43], or lack of health insurance coverage [44], were associated with higher prevalence of metabolic diseases. Finally, this supports previous observations that adverse SDoH can negatively impact health early in life [45–48].

While the HPI score captures a variety of SDoH, the greatest weight is placed on economic, education, and transportation factors. Other indices, specifically the SVI and CES, emphasize other SDoH. For this reason, we verified our findings among adults using the SVI and CES. As summarized in Table 3, we found the HPI explained the most variability in adult obesity while the SVI explained the most variability in adult diabetes and childhood obesity. This suggests that that the *Neighborhood & Built Environment* and *Social & Community* indicators represented in the HPI and SVI are important when considering how SDoH may impact metabolic health among children and adults, especially among Latinos. Collectively, these findings demonstrate that there is no “one size fits all” approach to examining composite indices as measures of SDoH. For this reason, future studies should consider the underlying indicators for a given composite index in the context of the research question and specific community under study.

A secondary aim of this analysis was to identify other health outcomes that were strongly associated with HPI, SVI and CES. This analysis revealed that a lower HPI score (more community-level adverse SDoH) was strongly associated with poor physical and mental health, lower life expectancy, and a greater prevalence of smoking, asthma, and heart attacks. Importantly, lower HPI scores and adverse health outcomes were observed in communities with a higher percent Latino population. These findings were consistent when examining the SVI and CES scores and agree with previous work that has shown that multiple adverse health outcomes can contribute to a shorter life expectancy, especially among vulnerable populations [16].

The current analysis examined the associations between composite indices of SDoH and metabolic health. Therefore, we were limited in our ability to infer causation or mechanistic explanations. Data completeness for health outcomes varied, ranging from 44% for adult diabetes and adult obesity to 93% for childhood obesity, making it impossible to perform a truly comprehensive analysis of southern California with these data. However, we found that the mean HPI, SVI, and CES scores was similar between all Southern California cities with and without available health data. City level HPI scores were based on a simple average of the HPI scores for census tracts assigned to a city. This calculation was performed because we wanted to examine the relative HPI scores across cities in Southern California. Future analyses could explore calculating a weighted city average for HPI score based on the population in each census tract. This analysis does not account for instances where an individual may live in a community with more adverse SDoH but commute to work in a community with less adverse SDoH. This is a common and generally accepted limitation of population-based approaches that analyze geographic areas rather than specific cohorts of patients. In other studies that follow defined groups of individuals, it is possible to account for exposures and time spent at school or work. Future studies should explore the impact of time spent outside one’s home census tract. In the current study, we utilized indices composed of indicators that are often highly correlated [23]. Thus, it is difficult to determine which aspects of the environment are most strongly associated with metabolic health. While we found that HPI, SVI, and CES were each strongly associated with certain health outcomes, we were unable to disentangle the relative contribution of individual-level factors. The inherent limitations of geocoded datasets and indices constructed from them also carry forward to this study. Finally, our focus on Latino communities limits the generalizability of our findings to other communities. Future investigations should explore associations between the HPI score with chronic disease prevalence among other communities of color as well as under-resourced and marginalized communities.

## Conclusions

Results from this study suggest that children and adults living in majority Latino communities in Southern California face a disproportionate burden of SDoH and experience higher prevalence of diabetes and obesity. Relative to more established measures of community vulnerability such as SVI, the HPI relies on a greater number of targeted SDoH indicators to compile a score that approximates the relative health conditions present in a community. All three indices capture adverse SDoH that contribute to disparities in metabolic health faced by communities of color, under-resourced, and marginalized communities. Future studies should explore how these composite indices can inform policies and strategies aimed at reducing the disproportionate burden of metabolic disease observed in Latino communities.

## Supplementary Information

**Additional file 1: Supplemental Table 1.** **HPI Domains and Indicators**. This table summarizes the domains and indicators presented in the HPI. The weights of the domains are presented, and the component indicators are classified according to their related SDoH domains. **Supplemental Table 2.** **SVI Domains and Indicators. **This table summarizes the domains and indicators presented in the SVI. The weights of the domains are presented, and the component indicators are classified according to their related SDoH domains. **Supplemental Table 3.** **CES Domains, Subdomains, and Indicators. **This table summarizes the domains, subdomains, and indicators presented in the CES. The weights of the domains are presented, and the component indicators are classified according to their related SDoH domains. **Supplemental Table 4.** **Health Outcomes.** This table presents the source documentation and description of the nine adult and the single child health outcomes analyzed. **Supplemental Table 5.** **Associations Between Population Weighted HPI and Simple Average HPI with Health Outcomes and Behaviors. **R^2^ values for the population weighted HPI and the simple average HPI are presented. The population weighted HPI explained a similar level of variability in the health outcomes of interest compared to the simple average of the HPI. **Supplemental Fig. 1. Comparison of Census Tract, City, and County Level Aggregation.** In Panel A, each point on the scatterplot represents a census tract within the ten counties investigated in this analysis. The x-axis represents the percent Latino within each of the Southern California census tracts. The y-axis represents the HPI Score Percentile, with 0 representing the census tract with the least healthy community conditions and 100 representing the community with the healthiest community conditions. In Panel B, each point on the scatterplot represents a city within the ten counties investigated. The x-axis represents the percent Latino within each of the Southern California cities. The y-axis represents the HPI Score Percentile, with 0 representing the city with the least healthy community conditions and 100 representing the community with the healthiest community conditions. In Panel C, each point on the scatterplot represents a county within the ten counties investigated. The x-axis represents the percent Latino within each of the Southern California counties. The y-axis represents the HPI Score Percentile, with 0 representing the county with the least healthy community conditions and 100 representing the community with the healthiest community conditions. **Supplemental Fig. 2. A Similar Negative Association is Observed in the SVI Score and Percent Latino.** Each point on this scatterplot represents a city in Southern California considered in the analysis. Cities are grouped into counties, the colors of which are depicted on the righthand legend of the graph. The x-axis is percent Latino for each of the Southern California cities considered in this analysis. The y-axis represents the SVI Score percentile, with 0 representing the community with the healthiest SVI score and 100 representing the community with the least healthy SVI score. Relative to the HPI, the SVI score is interpreted in the opposite direction, with 0 representing communities with lowest vulnerability. **Supplemental Fig. 3. A Similar Negative Association is Observed in the CES Score and Percent Latino.** Each point on this scatterplot represents a city in Southern California considered in the analysis. Cities are grouped into counties, the colors of which are depicted on the righthand legend of the graph. The x-axis is percent Latino for each of the Southern California cities considered in this analysis. The y-axis represents the CES Score percentile, with 0 representing the community with the healthiest CES score and 100 representing the community with the least healthy CES score. Relative to the HPI, the CES score is interpreted in the opposite direction, with 0 representing communities with the lowest vulnerability and lowest exposure to pollution burden. **Supplemental Fig. 4. SVI Demonstrates that Latinos Live in Less Healthy Community Conditions with Higher Prevalence of Disease.** Each point on the scatterplot represents a city within the ten counties investigated in this analysis. The x-axis shows the percentile SVI score, and a higher percentile score indicates a less healthy community. The y-axis shows the percentile score of the prevalence of each health outcome relative to each other (i.e., a percentile score of 100 translates to the city with the highest prevalence of adult obesity or diabetes. Each point (i.e., city) on the scatterplot is colored based on the percent Latino in each city. A red shading indicates a higher percent Latino compared to a purple shading. Panel A shows the relationship between adults with diabetes and SVI score. Panel B shows the relationship between adults with obesity and SVI score. The directionality of the SVI score is opposite to the directionality of the HPI score, where a higher score indicates a greater level of community vulnerability. **Supplemental Fig. 5. CES Demonstrates that Latinos Live in Less Healthy Community Conditions with Higher Prevalence of Disease.** Each point on the scatterplot represents a city within the ten counties investigated in this analysis. The x-axis shows the percentile CES score, and a higher percentile score indicates a less healthy community. The y-axis shows the percentile score of the prevalence of each health outcome relative to each other (i.e., a percentile score of 100 translates to the city with the highest prevalence of adult obesity or diabetes). Each point (i.e., city) on the scatterplot is colored based on the percent Latino in each city. A red shading indicates a higher percent Latino compared to a purple shading. Panel A shows the relationship between adults with diabetes and CES score. Panel B shows the relationship between adults with obesity and CES score. Note that the CES score has the opposite directionality as the HPI score. A higher CES score indicates greater vulnerability and greater exposure to pollution burden. **Supplemental Fig. 6. Young Latinos Live in Less Healthy Community Conditions and Face a Greater Burden of Obesity.** Each point on the scatterplot represents a city within the ten counties investigated in this analysis. The x-axis shows the percentile SVI and CES score, and a higher percentile score indicates a less healthy community. The y-axis shows the percentile score of the prevalence of childhood obesity relative to each other (i.e., a percentile score of 100 translates to the city with the highest prevalence of childhood obesity). Each point (i.e., city) on the scatterplot is colored based on the percent Latino in each city. A red shading indicates a higher percent Latino compared to a purple shading. Panel A shows the relationship between children with obesity and SVI score. Panel B shows the relationship between children with obesity and CES score. Note that the SVI and CES scores have an opposite directionality compared to the HPI score. A higher SVI or CES score indicates greater vulnerability or greater exposure to pollution burden.

## Data Availability

The combined datasets used and/or analyzed during the current study are available from the corresponding author on reasonable request. Individually, each of the datasets used in this study are publicly available through the websites listed below. California Healthy Places Index 2.0: https://www.healthyplacesindex.org/ *To access the 2.0 Dataset, please see the “Additional HPI Tools” tab on the “HPI Map” webpage.* California EnviroScreen 4.0: https://oehha.ca.gov/calenviroscreen/report/calenviroscreen-40 Social Vulnerability Index 2018: https://www.atsdr.cdc.gov/placeandhealth/svi/index.html California Department of Education 2019 Physical Fitness Test Results: https://www.cde.ca.gov/ta/tg/pf/pftresearch.asp

## Abbreviations

BMI: Body Mass Index
CES: California EnviroScreen 4.0
CDC: Centers for Disease Control and Prevention
HPI: California Healthy Places Index 2.0
NCATS: National Center for Advancing Translational Sciences
NIEHS: National Institute of Environmental Health Sciences
NIMHD: National Institute on Minority Health and Health Disparities
PFT: Physical Fitness Test
SDoH: Social Determinants of Health
SVI: Social Vulnerability Index
