# Supplementary material for: The impact of social determinants of health on obesity and diabetes disparities among Latino communities in Southern California
Source: BMC Public Health. 2023 Jan 6;23:37. doi: 10.1186/s12889-022-14868-1 (PMC9817265; doi:10.1186/s12889-022-14868-1)

Supplemental Table 1: HPI Domains and Indicators

Domain (weight)	**Indicators:** Description of Indicators	
Economy (32%)	**Poverty:** Percent of population exceeding 200% of federal poverty level	
	**Employment:** Percentage of adults 25-64 employed	
	**Median Household Income**	
Education (17%)	**Bachelor's Attainment:** Percent of adults over 25 with bachelor's degree or higher	
	**High School Enrollment:** Percent of teenagers 15-17 years of age enrolled in school	
	**Pre-School Enrollment:** Percent of children 3-4 years of age enrolled in pre-school	
Transportation (16%)	**Automobile Access:** Percent of households with access to car	**Active Commute:** Percent people 16+ commuting by walking, cycling, or transit
Social Environment (10%)	**Voting:** Percent of registered voters in 2012 general election	**Two Parent Household:** Children under 18 with two parents in household
Neighborhood (9%)	**Parks:** Percent of population living within 0.5 mile of park or similar open access	
	**Tree Canopy:** Population weighted percentage of area with tree canopy	
	**Supermarket Access:** Percent of urban population living within 0.5 mile of nearest grocery store and rural population within 1 mile of nearest grocery store	
	**Alcohol Outlets:** Percent of population within 0.25 miles of alcohol outlet	
	**Retail Jobs:** Retail jobs / acre	
Healthcare Access (5%)	**Insured Adults:** Adults aged 18-64 with insurance	
Housing (5%)	**Homeownership:** Percent of owner-occupied housing units	
	**House Repair:** Percent of households with kitchen infrastructure & plumbing	
	**Severe Ownership Cost Burden:** Percent of low-income homeowners spending more than 50% of income on housing	
	**Severe Rent Cost Burden:** Percent of low-income renters spending more than 50% of income on housing	
	**Crowded Housing:** Percentage of households 1 or fewer occupants per room	
Clean Environment (5%)	**Diesel PM Emissions:** Diesel PM emissions from on-road & non-road sources	**Ozone Concentration:** Daily max 8-hour ozone concentration
	**Drinking Water:** CalEnviroScreen 3.0 drinking water contaminant index	**PM 2.5 Exposure:** Annual mean PM2.5 concentration

This table summarizes the domains and indicators presented in the HPI. The weights of the domains are presented, and the component indicators are classified according to their related SDoH domains.

Supplemental Table 2: SVI Domains and Indicators

Domain (weight)	**Indicators:** Description of Indicators
Socioeconomic Status (25%)	**Poverty:** Percent of population exceeding 100% of federal poverty level
	**Unemployment:** Percentage of population over 16 that is unemployed and eligible for labor force
	**Per Capita Income:** Per capita income
	**No High School Diploma:** Percent of adults over 25 with less than high school education
Household Composition & Disability (25%)	**Persons Aged 65 & Older:** Percent of population aged 65 and older
	**Persons Aged 17 & Younger:** Percent of population aged 17 and younger
	**Civilian with a Disability:** Percent of population with a disability
	**Single Parent Household with Children under 18:** Percent of single parent households with children under 18
Minority Status & Language (25%)	**Minority:** All persons except white, non-Hispanic
	**Persons Who Speaks English Less than "Well":** Percentage of households where no person above 14 who speaks English well
Housing Type & Transportation (25%)	**Multiple Housing Units:** Percent of housing in structures with 10 or more units
	**Mobile Homes:** Percentage of housing that are mobile homes
	**Crowding Housing:** Percentage of households with more people than rooms
	**Automobile Access:** Percentage of households with no vehicle available
	**Persons in Group Quarters:** Percentage of persons in group quarters

This table summarizes the domains and indicators presented in the SVI. The weights of the domains are presented, and the component indicators are classified according to their related SDoH domains.

Supplemental Table 3: CES Domains, Subdomains, and Indicators

Domain (weight)	***Subdomains***	**Indicators:** Description of Indicators
Pollution Burden (50%)	***Exposures***	**Ozone:** Daily max 8-hour ozone concentrations
		**$PM_{2.5}$:** Annual mean concentration of PM 2.5
		**Diesel PM:** Gridded diesel PM emissions from on-road and non-road sources
		**Drinking Water:** Drinking water contaminant index
		**Children's Lead Risk from Housing:** Risk for lead exposure for children in low-income communities with older housing
		**Pesticide Use:** Total pounds of pesticide used per square mile
		**Toxic Releases from Facilities:** Toxicity-weighted concentrations of modeled chemical releases
		**Traffic Impacts:** Traffic volumes / total road length within 150 meters of census tract
	Environmental Effects	**Cleanup Sites:** Number of sites undergoing cleanup actions due to presence of hazardous substances
		**Groundwater Threats:** Number of sites that pose threat to groundwater within a census tract
		**Hazardous Waste:** Number of hazardous waste facilities and hazardous waste generators
		**Impaired Water Bodies:** Number of pollutants across impaired water bodies
		**Solid Waste Sites & Facilities:** Number of solid waste sites and facilities
Population Characteristics (50%)	***Sensitive Populations***	**Asthma ER Visits:** Emergency department visits for asthma per 10,000
		**Heart Attack ER Visits:** Emergency department visits for heart attack per 10,000
		**Low Birth-Weight Infants:** Percent of infants with low birth weight
	Socioeconomic Factors	**No High School Diploma:** Percent of adults over 25 with less than high school education
		**Low Income Household Severe Housing Cost Burden:** Low-income homeowners and renters paying more than 50% of income to housing
		**Persons Who Speaks English Less than "Well":** Percentage of households where no person above 14 who speaks English well
		**Poverty:** Percent of population exceeding 200% of federal poverty level
		**Unemployment:** Percentage of population over 16 that is unemployed and eligible for labor force

This table summarizes the domains, subdomains, and indicators presented in the CES. The weights of the domains are presented, and the component indicators are classified according to their related SDoH domains.

Supplemental Table 4: Health Outcomes

Data Source	**Outcomes:** Description of Outcome Specific Data
2014 CDC 500 Cities Project (162 / 367 cities had reliable data)	**Diabetes Prevalence:** Number of respondents 18 or older that self-reported being told by healthcare professional they have diabetes (other than diabetes during pregnancy)
	**Obesity Prevalence:** Calculated using self-reported BMI values from respondents 18 or older
	**Poor Mental Health Prevalence:** Adults 18 or older who self-reported that mental health was not good for 14 or more days over past 30 days
	**Poor Physical Health Prevalence:** Adults 18 or older who self-reported that physical health was not good for 14 or more days over past 30 days
	**Current Smoking Prevalence:** Adults 18 or older self-reporting they have either smoked more than 100 cigarettes in their lifetime or they currently smoke on some days
	**Asthma Prevalence:** Number of respondents 18 or older that self-reported being told by healthcare professional they have asthma
2011-2013 Cal EnviroScreen 3.0 (367/367 cities had reliable data)	**Asthma ER Admissions:** Rate of emergency department admissions for asthma, per 10,000
	**Heart Attack ER Admissions:** Rate of emergency department admissions for heart attack, per 10,000
2010 Virginia Commonwealth University Center for Society and Health (367/367 cities had reliable data)	**Life Expectancy at Birth**
2018 – 2019 California Physical Fitness Testing Data (341 / 367 cities had reliable data)	**Childhood Obesity:** Calculated using school-reported body composition results from respondents in the 5th, 7th, and 9th grades

This table presents the source documentation and description of the ten adult and the single child health outcomes analyzed.

Supplemental Table 5: Associations Between Population Weighted HPI and Simple Average HPI with Health Outcomes and Behaviors

**Health Outcome**	**R^2**	
	Population Weighted HPI	Simple Average HPI
Adults 18+ Who Report 14+ Days During Past Month Which Physical Health was Not Good	0.69	0.72
Adults 18+ Who Report 14+ Days During Past Month Which Mental Health was Not Good	0.65	0.67
Adults 18+ Who Report Having Smoked 100+ Cigarettes in Lifetime and Currently Smoke Every Day or Some Days	0.60	0.61
Adults 18+ With BMI ≥ 30.0	0.55	0.61
Age Adjusted Rate of Emergency Dept Visits for Asthma Per 10,000	0.47	0.49
Adults 18+ Diagnosed with Diabetes (Excluding Gestational Diabetes)	0.43	0.47
Percent of Population Currently with Asthma	0.45	0.46
Life Expectancy at Birth	0.41	0.42
Children with Obesity	0.38	0.41
Rate of ER Visits for Heart Attacks (Per 10,000 ER Visits)	0.38	0.40

R^2 values for the population weighted HPI and simple average HPI are presented. The population weighted HPI explained a similar level of variability in the health outcomes of interest compared to the simple average of the HPI.

Supplemental Figure 1: Comparison of Census Tract, City, and County Level Aggregation
In Panel A, each point on the scatterplot represents a census tract within the ten counties investigated in this analysis. The x-axis represents the percent Latino within each of the Southern California census tracts. The y-axis represents the HPI Score Percentile, with 0 representing the census tract with the least healthy community conditions and 100 representing the community with the healthiest community conditions. In Panel B, each point on the scatterplot represents a city within the ten counties investigated. The x-axis represents the percent Latino within each of the Southern California cities. The y-axis represents the HPI Score Percentile, with 0 representing the city with the least healthy community conditions and 100 representing the community with the healthiest community conditions. In Panel C, each point on the scatterplot represents a county within the ten counties investigated. The x-axis represents the percent Latino within each of the Southern California counties. The y-axis represents the HPI Score Percentile, with 0 representing the county with the least healthy community conditions and 100 representing the community with the healthiest community conditions.

Supplemental Figure 2: A Similar Negative Association is Observed in the SVI Score and Percent Latino. Each point on this scatterplot represents a city in Southern California considered in the analysis. Cities are grouped into counties, the colors of which are depicted on the right-hand legend of the graph. The x-axis is percent Latino for each of the Southern California cities considered in this analysis. The y-axis represents the SVI Score percentile, with 0 representing the community with the healthiest SVI score and 100 representing the community with the least healthy SVI score. Relative to the HPI, the SVI score is interpreted in the opposite direction, with 0 representing communities with lowest vulnerability.

Supplemental Figure 3: A Similar Negative Association is Observed in the CES Score and Percent Latino. Each point on this scatterplot represents a city in Southern California considered in the analysis. Cities are grouped into counties, the colors of which are depicted on the right-hand legend of the graph. The x-axis is percent Latino for each of the Southern California cities considered in this analysis. The y-axis represents the CES Score percentile, with 0 representing the community with the healthiest CES score and 100 representing the community with the least healthy CES score. Relative to the HPI, the CES score is interpreted in the opposite direction, with 0 representing communities with the lowest vulnerability and lowest exposure to pollution burden.

Supplemental Figure 4: SVI Demonstrates that Latinos Live in Less Healthy Community Conditions with Higher Prevalence of Disease. Each point on the scatterplot represents a city within the ten counties investigated in this analysis. The x-axis shows the percentile SVI score, and a higher percentile score indicates a less healthy community. The y-axis shows the percentile score of the prevalence of each health outcome relative to each other (i.e., a percentile score of 100 translates to the city with the highest prevalence of adult obesity or diabetes. Each point (i.e., city) on the scatterplot is colored based on the percent Latino in each city. A red shading indicates a higher percent Latino compared to a purple shading. Panel A shows the relationship between adults with diabetes and SVI score. Panel B shows the relationship between adults with obesity and SVI score. The directionality of the SVI score is opposite to the directionality of the HPI score, where a higher score indicates a greater level of community vulnerability.

Supplemental Figure 5: CES Demonstrates that Latinos Live in Less Healthy Community Conditions with Higher Prevalence of Disease. Each point on the scatterplot represents a city within the ten counties investigated in this analysis. The x-axis shows the percentile CES score, and a higher percentile score indicates a less healthy community. The y-axis shows the percentile

score of the prevalence of each health outcome relative to each other (i.e., a percentile score of 100 translates to the city with the highest prevalence of adult obesity or diabetes). Each point (i.e., city) on the scatterplot is colored based on the percent Latino in each city. A red shading indicates a higher percent Latino compared to a purple shading. Panel A shows the relationship between adults with diabetes and CES score. Panel B shows the relationship between adults with obesity and CES score. Note that the CES score has the opposite directionality as the HPI score. A higher CES score indicates greater vulnerability and greater exposure to pollution burden.

Supplemental Figure 6: Young Latinos Live in Less Healthy Community Conditions and Face a Greater Burden of Obesity.

Each point on the scatterplot represents a city within the ten counties investigated in this analysis. The x-axis shows the percentile SVI and CES score, and a higher percentile score indicates a less healthy community. The y-axis shows the percentile score of the prevalence of childhood obesity relative to each other (i.e., a percentile score of 100 translates to the city with the highest prevalence of childhood obesity). Each point (i.e., city) on the scatterplot is colored based on the percent Latino in each city. A red shading indicates a higher percent Latino compared to a purple shading. Panel A shows the relationship between children with obesity and SVI score. Panel B shows the relationship between children with obesity and CES score. Note that the SVI and CES scores have an opposite directionality compared to the HPI score. A higher SVI or CES score indicates greater vulnerability or greater exposure to pollution burden.

Supplementary Figure 1

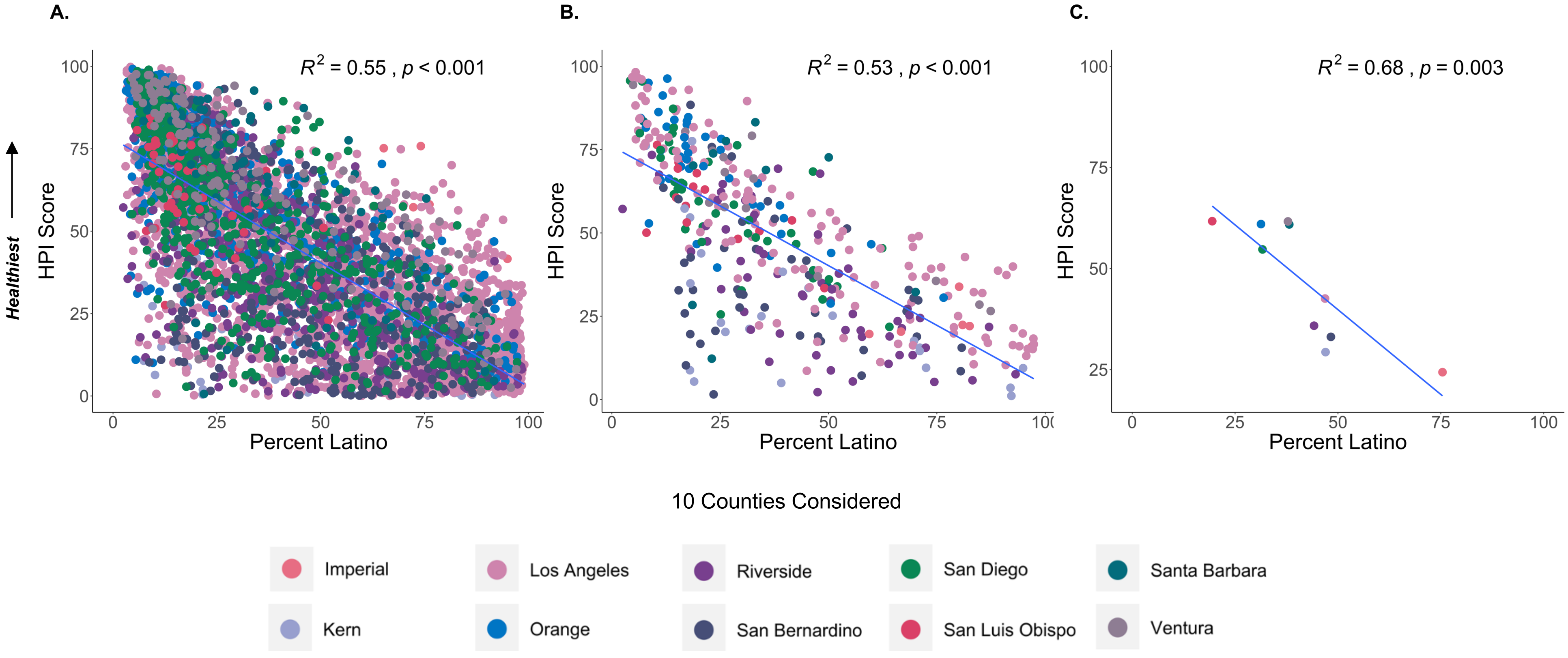

Supplementary Figure 2

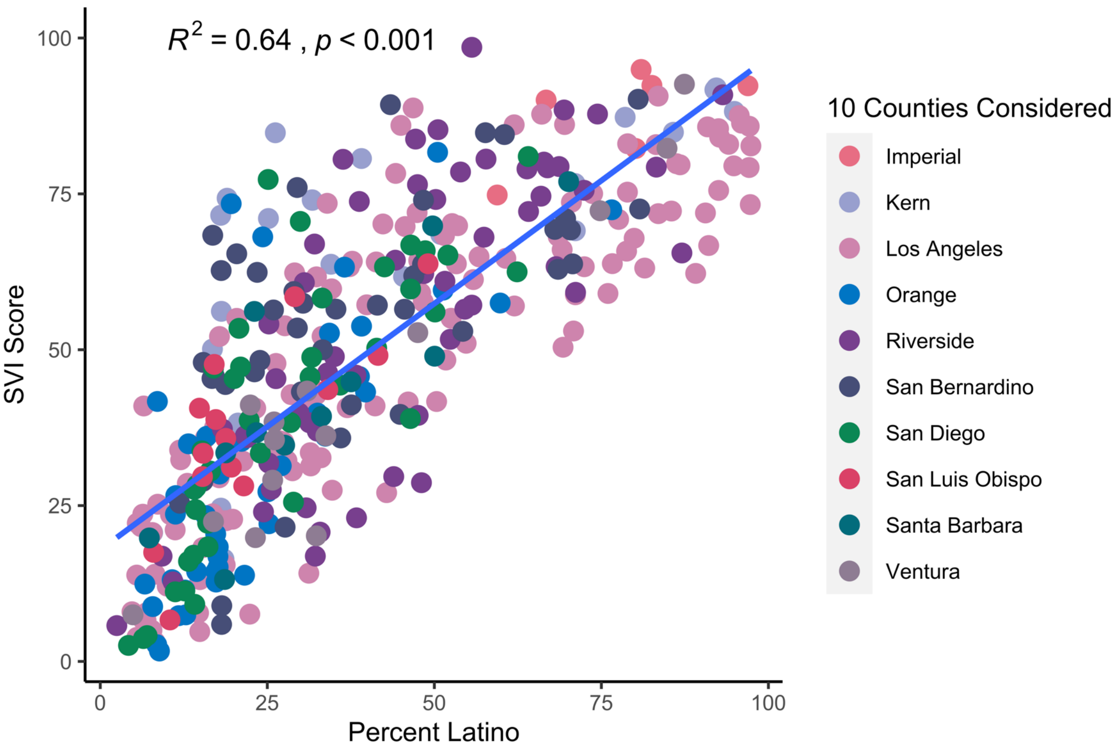

Supplementary Figure 3

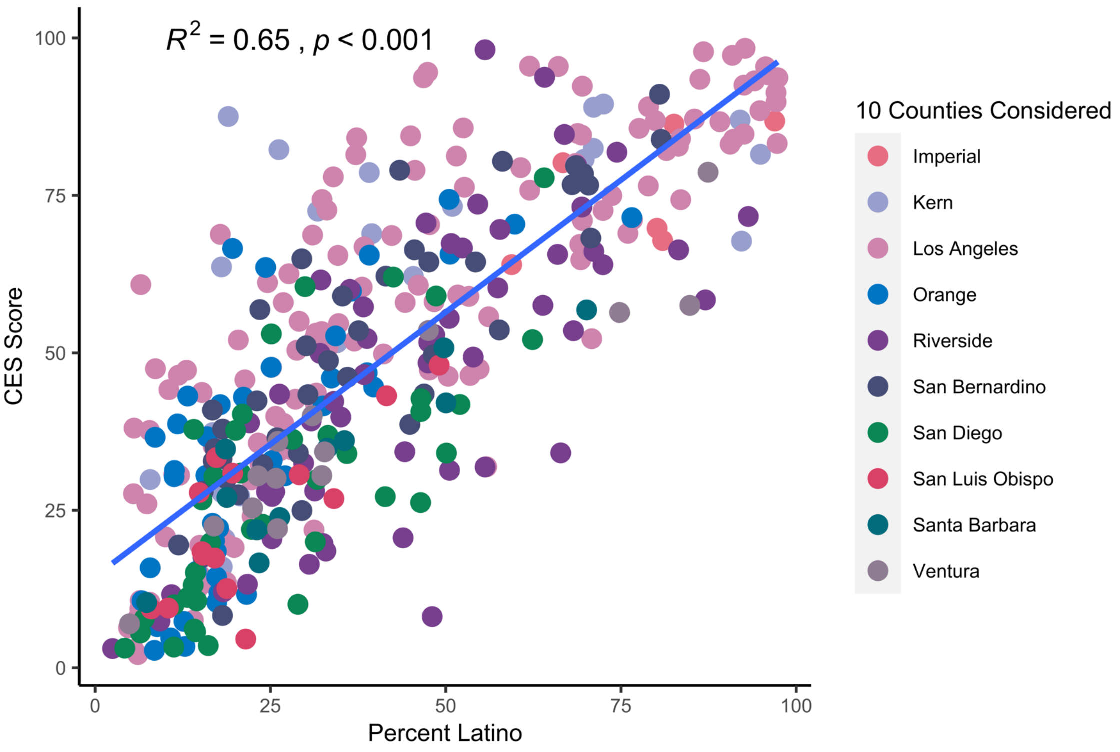

Supplementary Figure 4

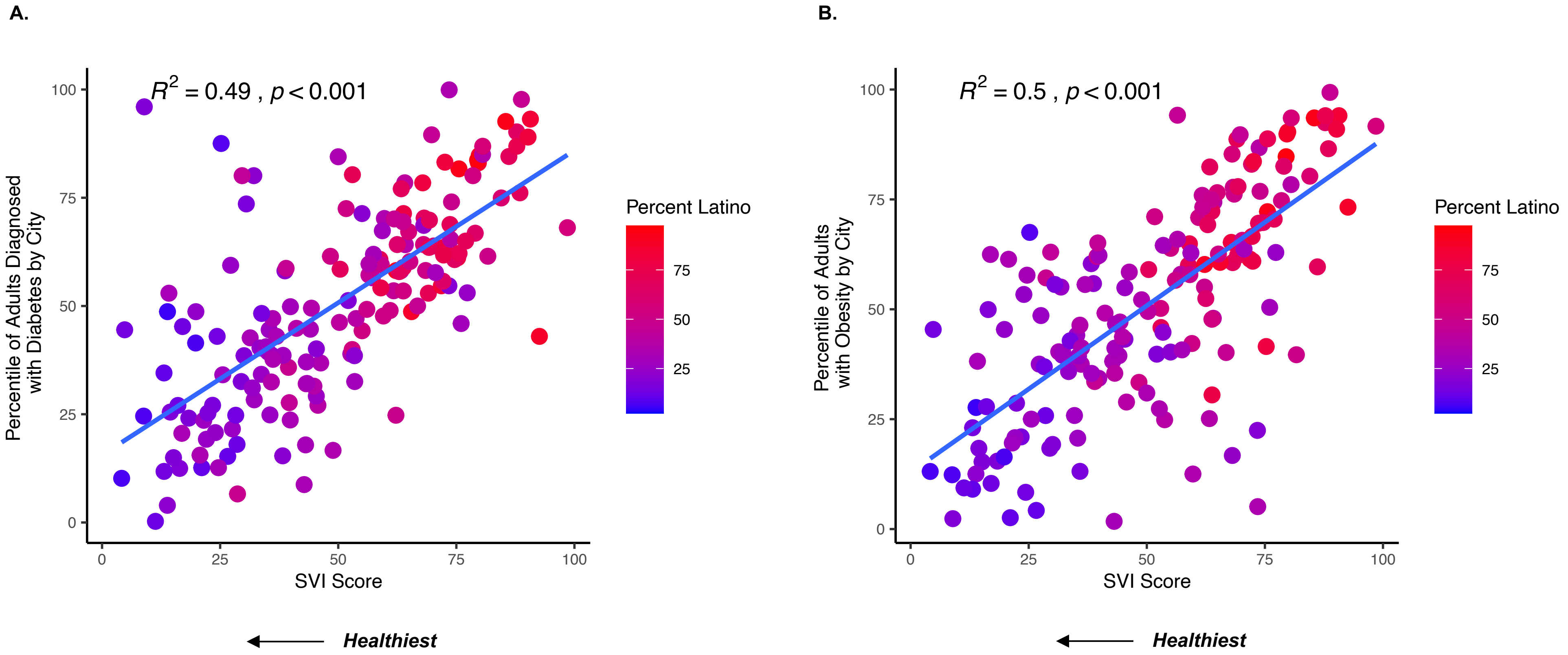

Supplementary Figure 5

A.

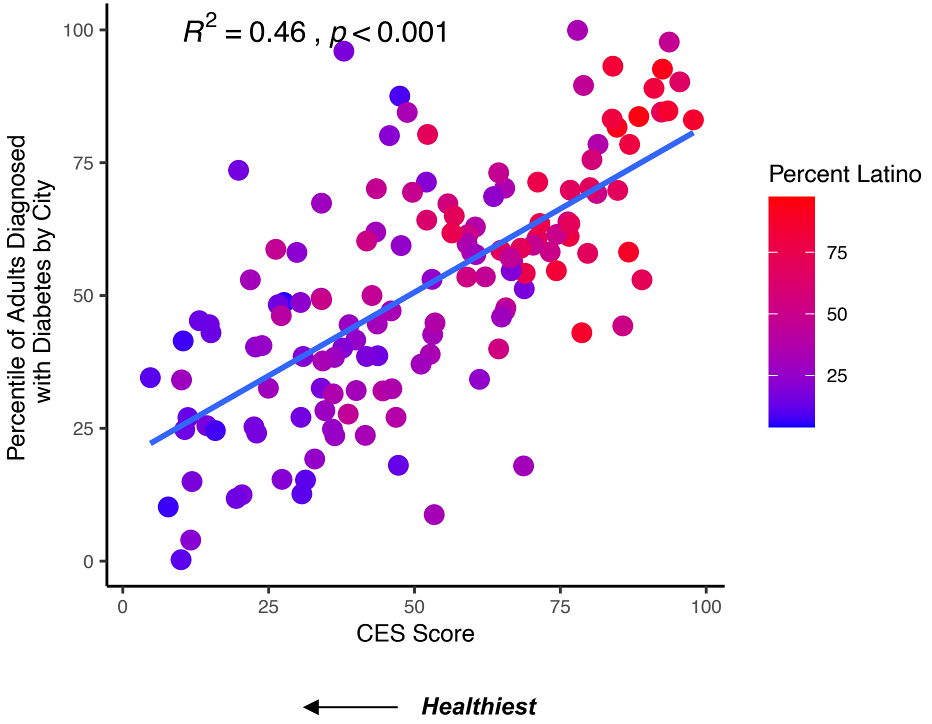

B.

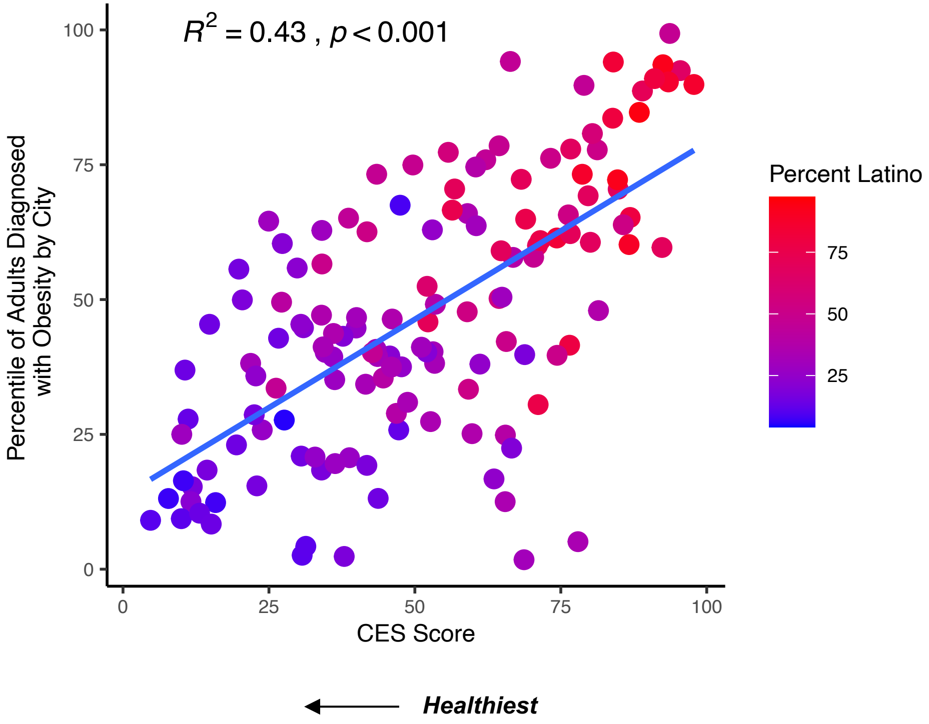

Supplementary Figure 6

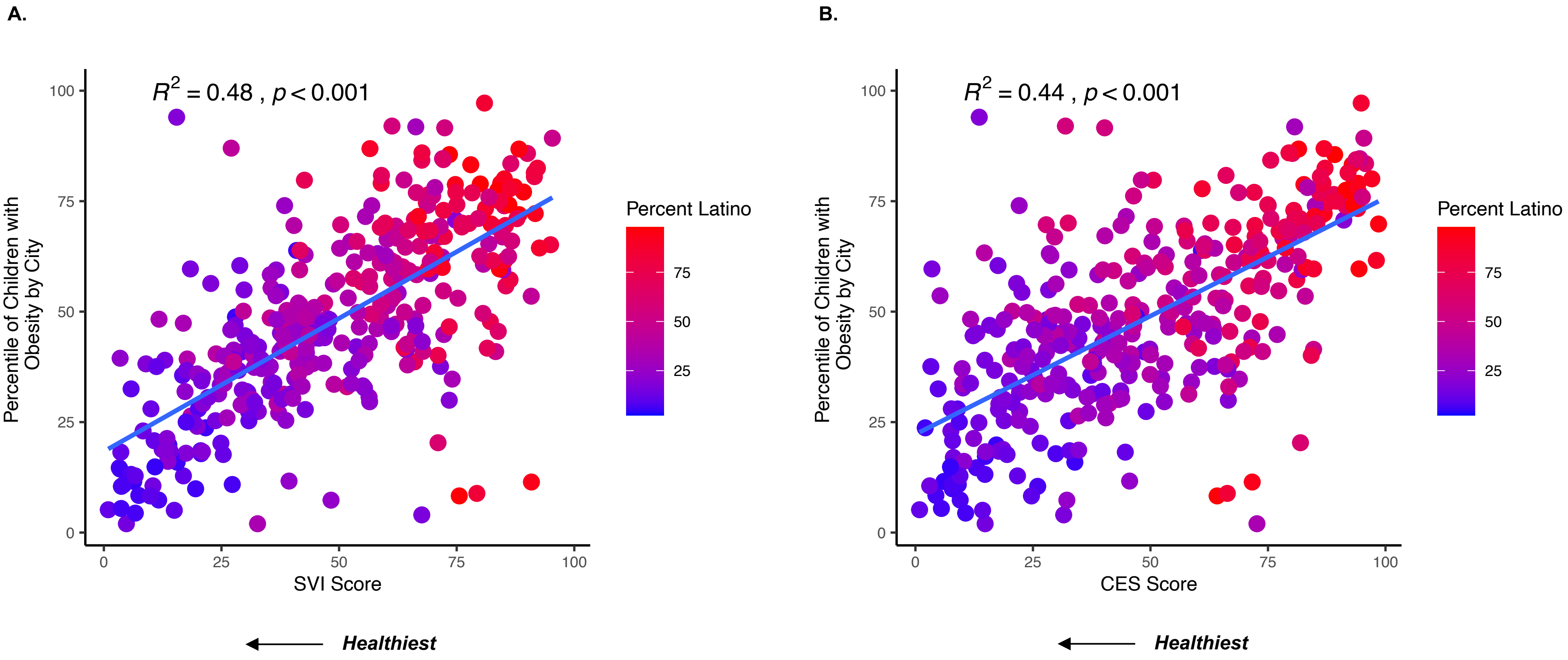

Supplement: Supplementary file 1 — Additional file 1: Supplemental Table 1. HPI Domains and Indicators. This table summarizes the domains and indicators presented in the HPI. The weights of the domains are presented, and the component indicators are classified according to their related SDoH domains. Supplemental Table 2. SVI Domains and Indicators. This table summarizes the domains and indicators presented in the SVI. The weights of the domains are presented, and the component indicators are classified according to their related SDoH domains. Supplemental Table 3. CES Domains, Subdomains, and Indicators. This table summarizes the domains, subdomains, and indicators presented in the CES. The weights of the domains are presented, and the component indicators are classified according to their related SDoH domains. Supplemental Table 4. Health Outcomes. This table presents the source documentation and description of the nine adult and the single child health outcomes analyzed. Supplemental Table 5. Associations Between Population Weighted HPI and Simple Average HPI with Health Outcomes and Behaviors. R2 values for the population weighted HPI and the simple average HPI are presented. The population weighted HPI explained a similar level of variability in the health outcomes of interest compared to the simple average of the HPI. Supplemental Fig. 1. Comparison of Census Tract, City, and County Level Aggregation. In Panel A, each point on the scatterplot represents a census tract within the ten counties investigated in this analysis. The x-axis represents the percent Latino within each of the Southern California census tracts. The y-axis represents the HPI Score Percentile, with 0 representing the census tract with the least healthy community conditions and 100 representing the community with the healthiest community conditions. In Panel B, each point on the scatterplot represents a city within the ten counties investigated. The x-axis represents the percent Latino within each of the Southern California ci [file 12889_2022_14868_MOESM1_ESM.pdf]
